# A Low-Cost Information Monitoring System for Smart Farming Applications

**DOI:** 10.3390/s20082367

**Published:** 2020-04-22

**Authors:** Muhammad Saqib, Tarik Adnan Almohamad, Raja Majid Mehmood

**Affiliations:** 1 Department of Computer Science, University of Engineering and Technology, Taxila 47050, Pakistan; saqib.khan5484@gmail.com; 2 Department of Electrical and Electronics Engineering, School of Electrical and Computer Engineering, Xiamen University Malaysia, Sepang 43900, Malaysia; tarikadnan.almohamad@xmu.edu.my or; 3 Information & Communication Technology Department, School of Electrical and Computer Engineering, Xiamen University Malaysia, Sepang 43900, Malaysia

**Keywords:** information monitoring, sensor, smart farming, wireless sensor network

## Abstract

A low-cost, low-power, and low data-rate solution is proposed to fulfill the requirements of information monitoring for actual large-scale agricultural farms. A small-scale farm can be easily managed. By contrast, a large farm will require automating equipment that contributes to crop production. Sensor based soil properties measurement plays an integral role in designing a fully automated agricultural farm, also provides more satisfactory results than any manual method. The existing information monitoring solutions are inefficient in terms of higher deployment cost and limited communication range to adapt the need of large-scale agriculture farms. A serial based low-power, long-range, and low-cost communication module is proposed to confront the challenges of monitoring information over long distances. In the proposed system, a tree-based communication mechanism is deployed to extend the communication range by adding intermediate nodes. Each sensor node consists of a solar panel, a rechargeable cell, a microcontroller, a moisture sensor, and a communication unit. Each node is capable to work as a sensor node and router node for network traffic. Minimized data logs from the central node are sent daily to the cloud for future analytics purpose. After conducting a detailed experiment in open sight, the communication distance measured 250 m between two points and increased to 750 m by adding two intermediate nodes. The minimum working current of each node was 2 mA, and the packet loss rate was approximately 2–5% on different packet sizes of the entire network. Results show that the proposed approach can be used as a reference model to meet the requirements for soil measurement, transmission, and storage in a large-scale agricultural farm.

## 1. Introduction

Smart farming represents the application of modern information and communication technologies to agriculture. It can potentially deliver productive and sustainable agricultural production based on a precise and resource efficient approach. Sensors in agricultural farms allow farmers to obtain detailed data in real time as variables, such as soil and ambient temperature, irrigation water and soil conductivity, and soil or irrigation water pH. With the use of communication technologies, these data can be transmitted to gateways to trigger the required actions based on soil properties and data logs, which can be sent to the cloud for future analytics [1,2].

To access field data with minimal wiring, easy installation, and low maintenance effort, different applications that utilize the distributed sensing features of wireless sensor networks (WSNs), including water monitoring [3,4,5], forest [6,7], industrial [8,9], agriculture [10,11,12], environmental [13,14], and smart city and community framework [15,16] have emerged. Such technologies are suitable platforms for implementing wireless systems in agriculture due to the application-oriented properties of WSNs. A WSN is a collection of nodes that work cooperatively. Each node includes a microcontroller, a power source, and a communication unit, and can accommodate multiple sensors. Sensor data are transferred to a gateway using the communication unit via single or multiple hops.

WSNs are emerging as a great aid to improve agriculture quality, productivity and resource optimization. At present, substantial research [17,18,19,20] has focused on developing efficient WSN systems that will provide fine-grain monitoring and automation of farming processes. The measured values of soil sensor nodes should be reliably transferred to a gateway via a communication medium. WSNs can collect data from field sensor nodes with low cost, minimal wiring, easy installation, and improved maintenance. A WSN-based network comprises end nodes for fetching field data measurement (e.g., temperature and moisture), a communication module for data transmission (e.g., ZigBee), and a central controller to manage sensor data, trigger actuators, and store data [21,22].

Currently, the most widely used ZigBee, Bluetooth, Cellular and other communication technologies have their own pros and cons [23,24]. In one system, the collected field environmental parameters e.g., moisture, temperature are used to send via ZigBee module toward sink node and a GPRS module is integrated into sink node for long distance communication with server which realized centralized information monitoring, data display, data storage and performs data analytics in the greenhouse [25]. In a traffic information detection system, Bluetooth is used to transmit the vehicle parameters e.g., position and speed etc. [26]. A GPRS based sensor monitoring system is proposed to ensure the accurate data transmission over long distance in a distributed environment [27]. However, ZigBee and Bluetooth are short-range radio technologies and are not suitable for long-range transmission scenarios. 2G, 3G, 4G, and other solutions based on cellular communication can provide a wider coverage, but they consume too much energy and increase the operating costs [24].

The core objectives of building the target system are distance coverage, cost effectiveness, and communication reliability. In this work, a low-cost communication module called HC12 is used for data transmission at long distance in the agriculture farm. HC12 has 200–1000 m communication range in point to point scenario. However, compared with the proposed system, cellular communication and LPWAN based solutions [24,28] provide better communication range over several kilometers in open sight. Nevertheless, a tradeoff exists between distance coverage and node placement in each zone. For example, if node placement distance increases in kilometers for the sake of a higher communication range, then a farmer may be unable to measure intermediate zones. By contrast, if node placement distance decreases, then deploying a costly system is unnecessary. In our application, zone coverage and distance coverage are equally important. To take the application requirements into accounts, HC12 has been considered the most appropriate solution. A network mechanism has been designed to extend the communication by adding intermediate nodes. To the best of our knowledge, the proposed work can best fit into application requirements.

The rest of this paper is organized as follows: The existing IoT technologies in information monitoring are discussed in Section 2. The key component and proposed technology are introduced in Section 3. The detailed methodology of the proposed system is discussed in Section 4. A set of real time experiments are presented and commented in Section 5. Finally, the conclusions, limitation and future work are summarized in Section 6.

## 2. Literature Review 

The role of WSNs in agriculture has become prominent as part of the precision agriculture (PA) initiative, and these networks help constitute PA [1,17]. The adaptation of WSN systems in agriculture has been widely explored in past decade [22,29] from different perspective e.g., design of wireless platform for better performance, optimal deployment strategy of sensor nodes and automated irrigation management systems for water saving. In [30], the parameters of IEEE 802.15.4 MAC layer are properly tuned to follow the sampling frequency of sensor nodes according to the requirements of precision farming. A quality sensor node deployment pattern is proposed to the precision agriculture [31]. The method uses several metrics for quantifying sensor deployment patterns to provide qualitative connectivity in the farm. From the cost perspective, the problem of optimal deployment is achieved by maintaining the desired level of coverage and connectivity with a minimum number of nodes [32]. Furthermore, the management of irrigation system is extensively analyzed to gain water saving [22,33,34]. For instance, the real time value from root zones of the plant are measured in a distributed manner and a threshold value used at the gateway to achieve water saving [21]. Recently, a decision support system (DSS) embedded in the network gateway outperformed the state-of-the-art methods based on parameters thresholding [22]. 

As a matter of fact, most of the effort has been done on the design and deployment strategies for WSN with the realization of sensing capability and better management in precision agriculture. Whereas, the reliable real time data transmission acquired by networked sensors over long distance has been less investigated. Although, many researchers have focused the attention on study and design of data transmission in WSN environment, but the common drawbacks are either its limited distance coverage or higher deployment cost. For example, ZigBee has always been considered an optimal solution because of its low power consumption and strong mobility [35,36,37]; however, its data transmission range is limited to 100 m between two points. The count of ZigBee devices increases when more than 100m are required to be covered; hence, implementation cost and network overhead will also increase. To ensure long distance coverage, a GSM-based solution is used wherein each node contains a GSM module and directly sends its sensor data to the cloud, thereby changing network topology from centralized to distributed [38]. Although coverage tension is eliminated, implementation cost and installation complexity are extremely high. Recently, a LoRa-based lowpower wide-area network (LPWAN) solution has been proposed to solve the information monitoring problem over a wide area [28]. A LoRa communication module provides reliable data transmission at a distance of more than 1 km in a relatively complex environment; however, the major drawback of the LPWAN solution is that it requires an annual subscription from a single vendor (Semtech) [39] and a dedicated gateway called NB-IoT/LoRaWAN [24], which may be costly.

One of the current challenge is to design a cost-effective system for the field data transmission over long distance, which is more desirable in this application, is rarely explored. Thus, the current study proposes and deploys a low-cost wireless sensor network, which can be used as a reference model for data collection from field sensors over a wide area. Nevertheless, some WSN-based information monitoring systems are already available [22,28,34,38], but the common drawback of these systems is the extremely high deployment cost when the count of devices increases.

## 3. IoT-Based Model Farm

**Figure 1:**
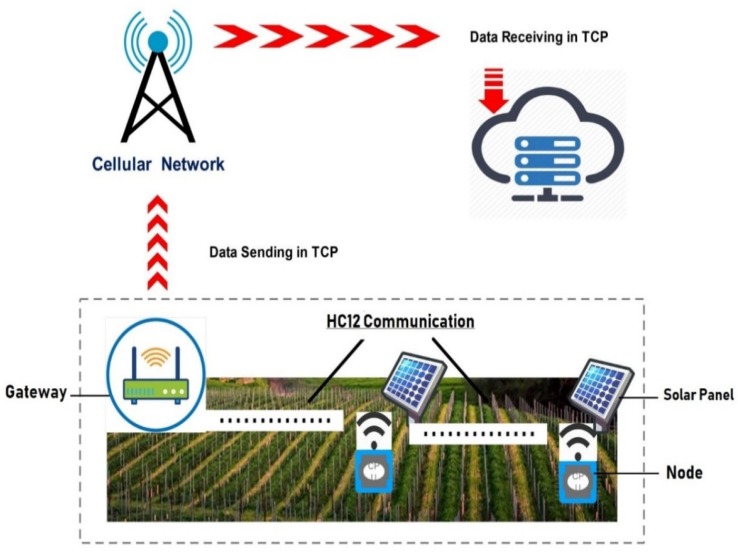
Block diagram of IoT-based agriculture farm.

Our proposed solution is application-specific and consists of moisture sensors, microcontrollers, radio frequency communication modules, solar panels, a Linux-based gateway, an Internet connectivity module, and cloud storage. The diagram of the proposed system design is shown in Figure 1. The solution is designed with the ultimate goal of ensuring high distance coverage with minimal deployment cost.

### 3.1. Overview of HC12

HC12 [40] is a new-generation half-duplex wireless transmission module, which has a frequency range of 433.4–473.0 MHz. The module has embedded multichannels and can use 100 channels with a stepping of 400 KHz. The maximum transmission power of the module is 100 mW (20 dBm), the receiving sensitivity is −112 dBm at a baud rate of 9600 in air, and the communication distance is 200–1000 m in open space. The farthest communication range can be achieved when a module is set to a low data rate. Three working modes, called FU1, FU2, and FU3, can be set to adapt to different application requirements. FU1 and FU2 are power-saving modes, whereas FU3 is the full power mode.

### 3.2. Overview of Orange-Pi

Orange PI is an open-source single-board computer that can operate on 5 V power. It has 512 MB SDRAM and 2 MB onboard serial peripheral interface (SPI) flash and can support a maximum of 32 GB TF card. Orange PI has up to 26 general purpose input/output (I/O) pins, which can be used for several purposes, two USB 2.0 slots, one SPI, one I2C, three universal asynchronous receiver transmitters (UARTs), and a hardware real-time clock/calendar. The microcontroller is suited well for this remote application and can run Android 4.4, Ubuntu, Debian, and Raspbian operating systems (OSs).

### 3.3. Overview of 2G Module (SIM900)

The 2G module is a GSM-based module that can deliver 850/900/1800/1900 MHz performance for voice and data. The module communicates with the main board using AT commands via a UART serial interface. The operating voltage range is 4.5–5.5 V. In this study, this module is used to collect weather data from the Internet and establish connection with the cloud using the GSM/GPRS protocol. Compared with 3G or 4G, the 2G module is more suitable for agricultural applications because most agricultural farms are located in the countryside, where 3G or 4G are not yet established. Although the data rate is considerably slower than the latest technology, speed is not an important factor in our context because the amount of collected data is extremely small.

## 4. System Design and Implementation

Our system primarily includes nodes (installed in the field), the central node (gateway), and cloud storage, as presented in Figure 1. Field data are measured using the sensor node’s attached sensor and are transmitted to the gateway using an HC12 communication module on single or multi hops. The central node receives field data from the sensor nodes, fetches weather data from Open Weather API, and sends minimized logs to the cloud using the 2G (GSM/GPRS) module.

### 4.1. Hardware Design

The layout of the nodes and the gateway has been designed by observing an application scenario, e.g., optimal data collection, low power consumption, and reliable transmission. 

#### 4.1.1. Hardware Design of Sensor Node

**Figure 2:**
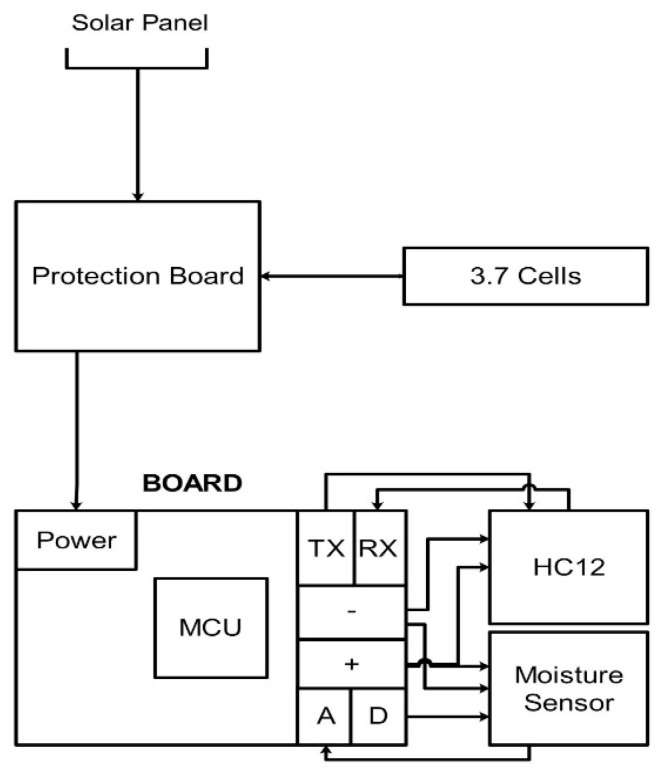
Hardware design of sensor node.

The basic job of a node is to receive a timely request from the gateway via a communication unit, collect field soil parameters, and send the resultant values to the gateway in the reply packet. Each node comprises a microcontroller, a soil moisture sensor, an HC12 communication unit, and a solar based power unit. A block diagram of a node is shown in Figure 2.

The microcontroller unit (MCU) is ATmega328p (Arduino Nano 3.0). The recommended input voltage for the microcontroller is 7–12 V, and the operating voltage (logic level) is 5 V. The MCU has 14 digital I/O and 8 analog input pins. Each pin has a DC current of 40 mA. The sum of all currents going into or out of the input/output pins (all analog and digital pins combined) of the ATMEGA328P microcontroller itself cannot exceed 200 mA. In our scenario, the sensor node has utilized 4 GPIO pins (3 digitals and 1 analog) which comes under the maximum limit of ATMEGA328P. The board flash memory is 16 KB and 2 KB SRAM. The soil moisture sensor is connected to analog and digital pins on the microcontroller board. The microcontroller controls the sensor power using the digital pin to keep it low during idle times. When moisture request is received from the gateway, an interruption is generated to trigger the digital pin voltage, i.e., high for a short interval and then returns to low, to reduce power consumption. 

The HC12 hardware consists of a built-in MCU, a TTL serial communication interface, a power supply, a mode control, and an antenna. The built-in MCU communicates with an external device using the serial port. HC12 can be powered by 3.2–5.5 DC voltage. Data transmission has three modes, namely, FU1, FU2, and FU3, which can be set using AT commands in accordance with application requirements. 

In this study, we only use the FU3 mode, which has an average power consumption of 16 mA (in idle state) and the maximum current consumption is measured between 50–55 mA (in transmission state). Two paired modules must have the same transmission mode, serial baud rate, and wireless communication channel. Furthermore, the module is half duplex, and data cannot be sent and received simultaneously between two modules. 

**Figure 3:**
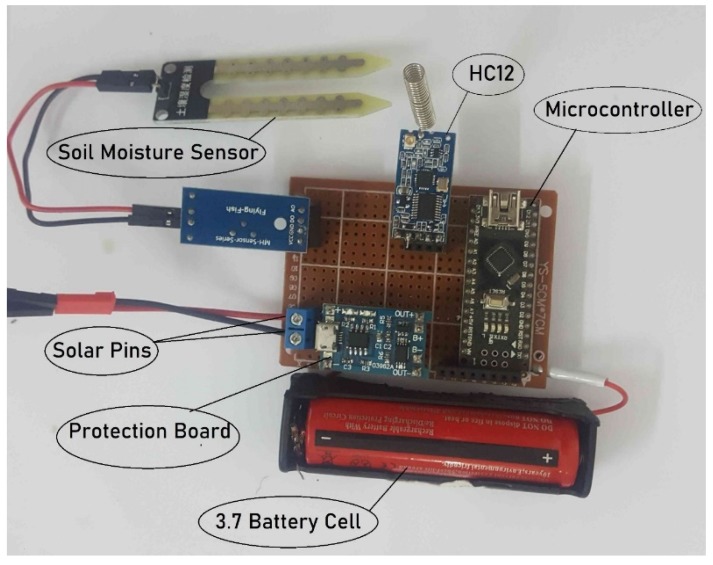
Physical diagram of sensor node.

The power unit consists of a 10 W solar panel, a battery protection board, and a 3.7 storage cell. The protection board is used to regulate the voltage output of a solar panel and to prevent the charging cell from being overcharged. The 4 V output voltage goes directly to the microcontroller, soil sensor, and communication unit. The storage cell is charged during the day, which keeps the sensor node alive in partially cloudy weather, even at night. The node’s operating life was estimated by measuring the actual current consumption. For current measurement experiment, we programmed the sensor node to continuously measure the soil moisture status and transmit the resulting values to the gateway, continuously. While running the device in full working mode, the measured current consumption of sensor node was 80–85 mA. The current consumption of individual module e.g., microcontroller, sensor and transceiver were measured as 20 mA, 5 mA and 55 mA, respectively. For the operating life measurement, the sensor node was powered-up by a fully charges 3.7 V cell with a capacity of 1800 mAh. As the device current consumption was 80–85 mA, the battery was lasted for almost 20 h in the experiment. Physical diagram of the sensor node is shown in Figure 3.

#### 4.1.2. Hardware Design of Gateway

**Figure 4:**
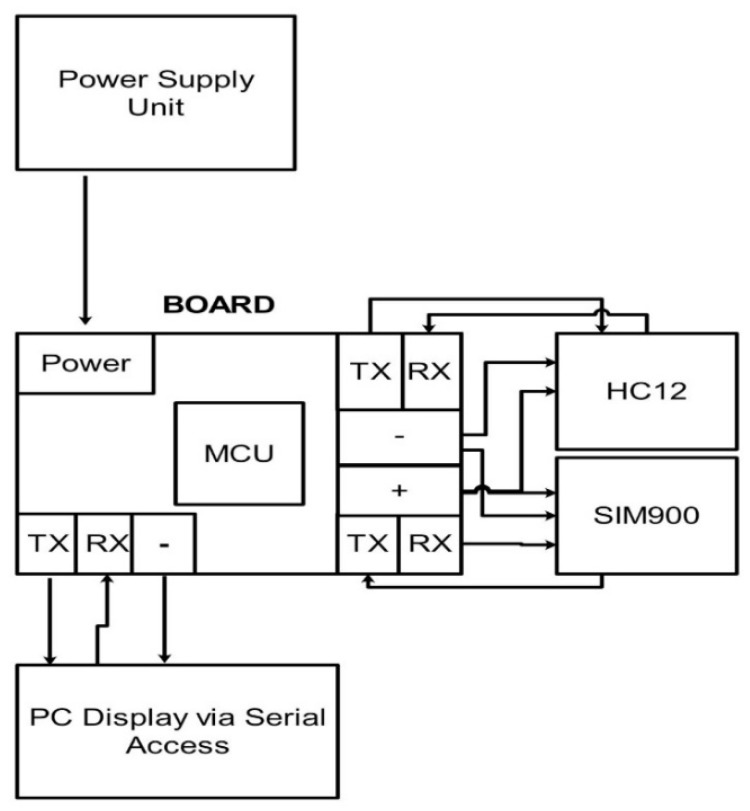
Hardware design of gateway node.

**Figure 5:**
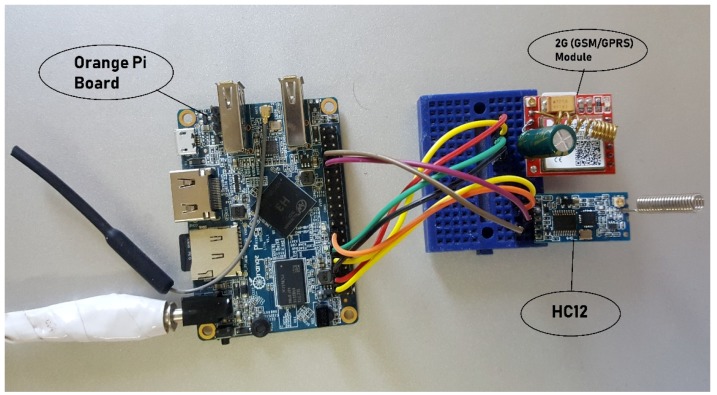
Physical diagram of gateway node.

The gateway is dedicated to collecting field data from all nodes and weather data from the Internet and to send minimized data logs to the cloud storage. An Armbian based device, called Orange Pi, is used for operating purposes. The device is installed at a central location in a farm where electricity is available, thereby eliminating the need for a solar panel and a protection board. The hardware design of the primary node comprises a 5 V DC power supply, an Orange Pi device, a 2G (GSM/GPRS) module, and an HC12 communication unit. The HC12 and GPS modules are connected to the Orange Pi board on serial interfaces. The block diagram of the central node is shown in Figure 4, whereas the physical diagram is shown in Figure 5.

### 4.2. Software Design

The software design of the proposed system includes a tree-based network communication mechanism embedded into nodes and a gateway program. A single communication unit cannot satisfy the requirements for a large-scale farm due to its limited range. Therefore, the complete logic of the network is implemented on the software side of the nodes and the gateway. 

#### 4.2.1. Software Design of Sensor Node

The software design of nodes includes a configuration function, a main loop, a data collection function, and network logic. The compilation and development of the complete program are performed using C++ language with Arduino software. In the configuration part, we set input pins for the sensor, a serial baud rate, and HC12-related commands, which include the transmission mode, the baud rate, and the wireless transmission channel. A SET pin is available on the communication unit, which should be set to ground at configuration time.

**Figure 6:**
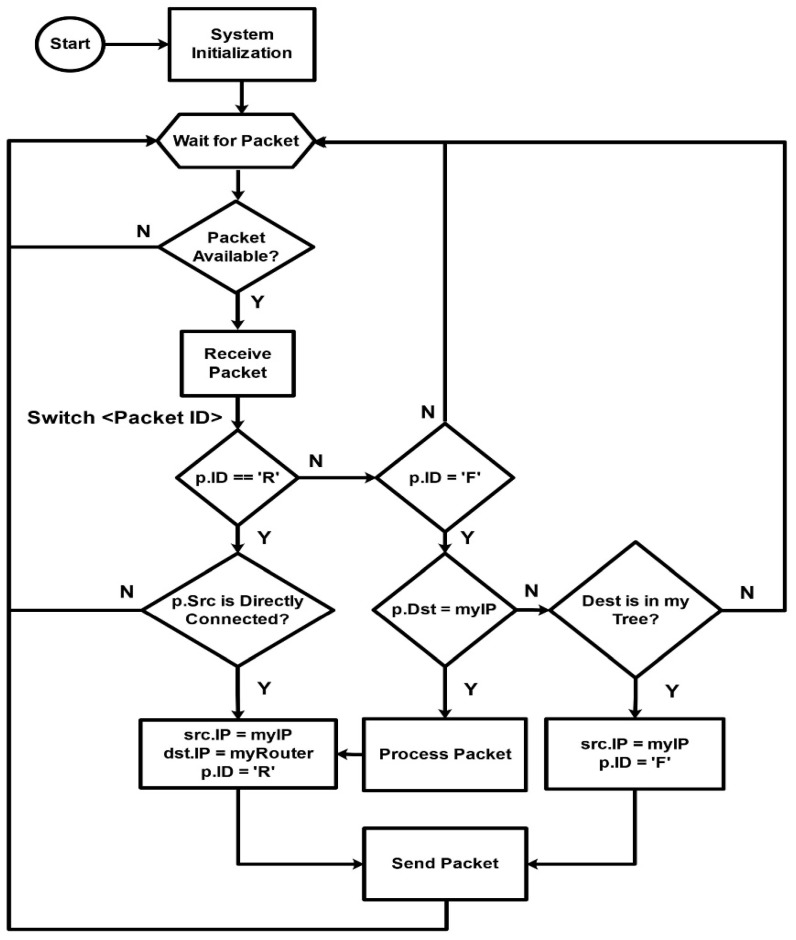
Flow diagram of sensor node.

Configuration can be run at each reset. In the main program, a loop is always waiting for incoming packets. If an incoming packet is destined for that node, then the sensor pin is triggered to collect field data and send the resultant value in the reply packet to the gateway. The complete flow diagram of nodes is shown in Figure 6, in which a node is used to forward the packet if the destination address does not match its address. In this study, we only use the moisture sensor, which collects data thrice and then takes the average to obtain accurate results.

#### 4.2.2. Software Design of Gateway

**Figure 7:**
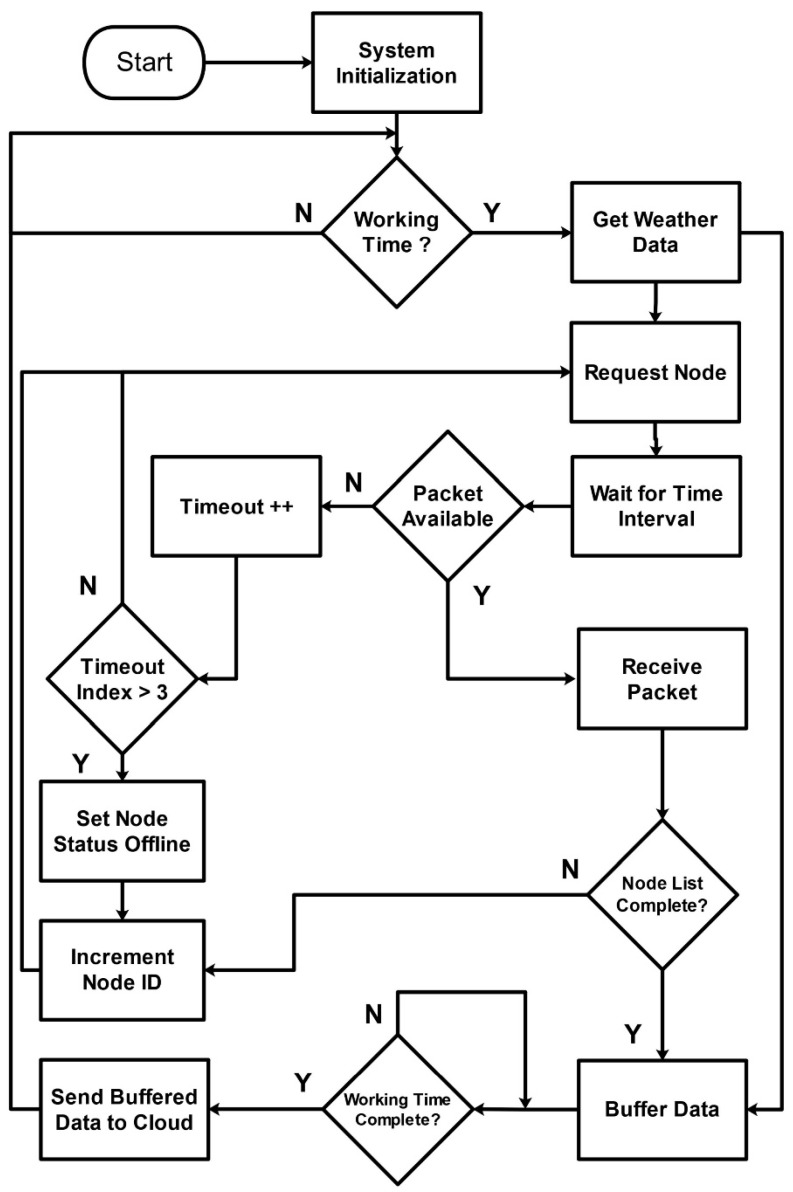
Flow diagram of gateway node.

The software design of the gateway is developed based on the hardware design, which primarily includes configuration, data collection, and data storage. The compilation and development of the complete program is performed using the Python language on the Raspbian OS Image. The flow diagram of the gateway is shown in Figure 7. 

The configuration of the central node includes AT commands for the communication unit and the GSM module and login credentials for cloud storage. After initial configuration, the data collection process starts to run daily at morning to collect field and weather data. This process also uses a retry mechanism to overcome packet loss due to connectivity issues between nodes. At each node request, the response is stored locally with a node ID or the node ID is added to the dead list after time-out. After data completion from all nodes and the Open Weather API, the gateway starts retransmission process for offline nodes using its neighbor nodes. Finally, to save the bandwidth, the gateway starts data minimization process on locally stored data, to send the minimized logs to the cloud. 

### 4.3. Network Architecture

**Figure 8:**
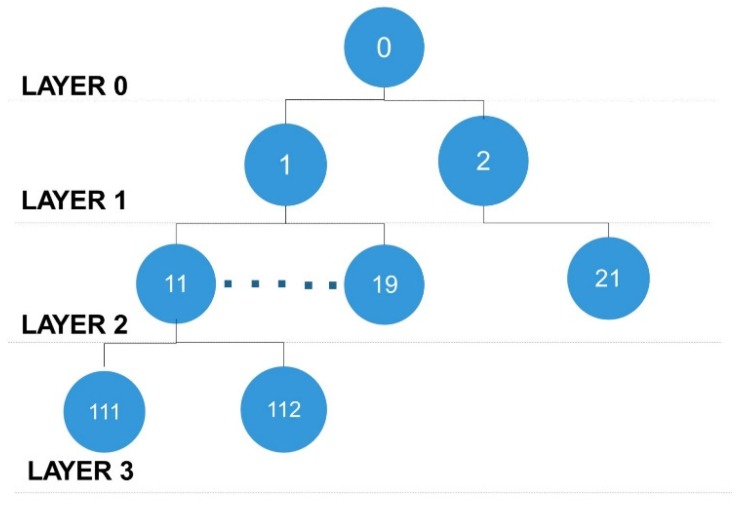
Layered based network architecture.

The network mechanism is designed based on a tree topology. The communication module range is limited; thus, distance coverage is extended by adding intermediate nodes. A simple layer-based approach is used where each intermediate node creates a layer. The count of layers increases with the count of intermediate nodes. The lower layer node depends on the liveness of the upper layer node, where the failure of one node may cause the entire subnetwork to become unavailable. Each node can host one to nine directly connected nodes and may have many indirectly connected nodes, as shown in Figure 8.

The gateway node is located at the top layer, which generates data request, whereas all the sensor nodes are found at the lower layers. When the packet originates from layer 0 (gateway) toward the targeted nodes, each node checks if the packet is destined for it and then processes the packet; otherwise, it forwards the packet to its child nodes. To overcome unnecessary packet forwarding and network overhead, the address length is defined at each layer of the tree (e.g., layer 1 has a single-digit address, layer 2 has two digits, and layer 3 has three digits of the address). The length of the destination address at each node is used to calculate in digits prior to packet forwarding. For example, a packet is generated with the targeted address 112, and node 1 will forward the packet to all nodes in its subtree. When the packet is received at the second layer, node 11 forwards the packet to its sensor nodes, whereas the other nodes will immediately drop the packet. The network logic for packet process and movement is implemented at the sensor node.

Wireless sensor networks (WSNs) consist of spatially distributed autonomous sensor nodes to cooperatively monitor certain events and phenomena in an interesting area. As compared to wired network, the WSN’s nodes are very to link failure due to their limited available resources [41]. The failure also impacts the sub network if the underlying topology is tree based. Such failure not only cause to coverage loss of monitored area but also disjoint some nodes with the base station. Therefore, it is crucial to restore connectivity from such damaged WSNs.

**Figure 9:**
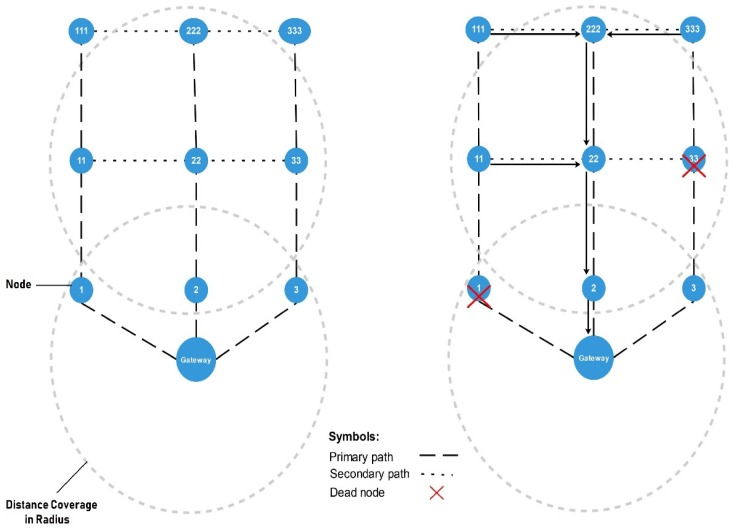
Retransmission diagram for offline nodes.

Connectivity restoration issue can be resolved by placing relay node (RN) [42,43]. In our system, each node is designed to act as a sensor node and a relay node as well. With the realization of connectivity restoration, we classified the neighbor nodes into primary and secondary paths, to re-route the traffic in case of failure. All the nodes are considered neighbors which are placed within a radius and can communication directly. Each node has four neighbors in its radius: column-wise, which follows primary path vertically and row-wise, which follows secondary path horizontally. In case of any failure at primary path’s node, the gateway re-routes the traffic to secondary path as shown in Figure 9. The node marks as dead if it fails to response in time interval. 

A 10-byte custom packet structure is developed, as shown in Table 1. Three types of packet ID (S, F, and R) are used in our packet structure. When a packet originates from the gateway, the packet ID is set to S (status packet). Packet IDs F and R are used for forwarding and replying purposes, respectively.

## 5. Results and Discussion

An experimental test is performed in a line of sight on a large-scale grape farm, which covers nearly 700 m from east to west and 500 m from north to south. The real-time test aims to measure the communication range, the transmission delay time, and the packet success rate in the network on a different packet size. Soil moisture sensors are used to measure the moisture level in different areas of the farm to validate the results because moisture value is familiar to farmers.

### 5.1. Point-to-Point Communication Distance Test

**Figure 10:**
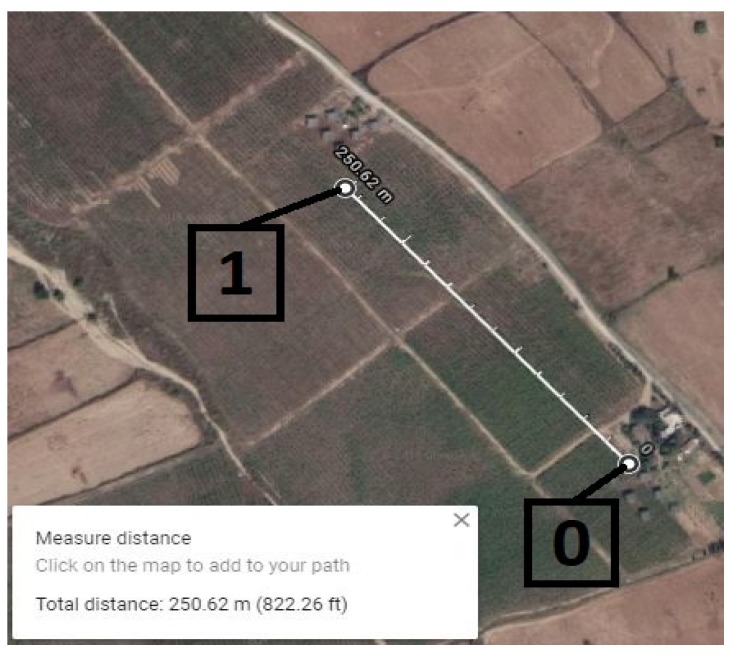
Point-to-point communication distance map.

The first step is to measure the communication range between two directly connected nodes. The central node (gateway) is fixed on the east–north corner, which generates data request for sensor nodes in a timely manner. The response rate at the gateway is examined by generating a continuous data request and slowly moving node 1. The optimal measured distance is approximately 250 m, as shown in Figure 10. To measure packet loss, 1000 beacon packets are sent from the gateway toward node 1, which results in a packet loss rate of 1.5% between two directly connected nodes.

### 5.2. Multi-Hope Communication Distance Test

**Figure 11:**
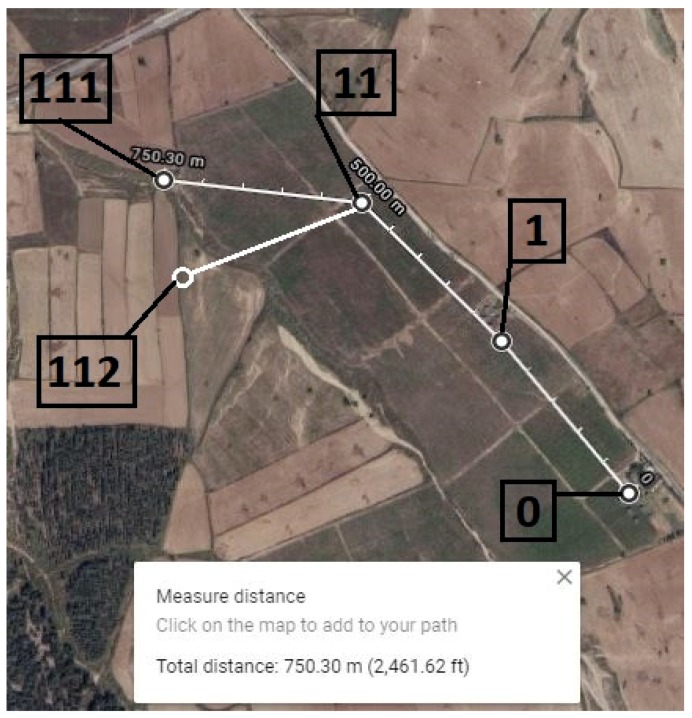
Multi-hope communication distance map.

The actual work of the network is examined via a multi-hop communication distance test, in which each node can serve as a router and a data collection unit. Considering the previous result, node 1 was initially fixed in position #1 from where it was responding reliably in the point-to-point test. As a mobile node, node 11 moved slowly, the response rate was measured continuously, and node 11 was fixed in position #11 at a distance of 250 m next to node 1. In addition, two more nodes (111 and 112) were deployed next to node 11 to check the work of multiple nodes at the same layer. The last two nodes were deployed in opposite directions at a distance of 250 m from node 11 in positions #111 and #112, as shown in Figure 11.

As shown in the network architecture, the gateway, node 1, and node 11 are found at layer 0, the first layer, and the second layer, respectively; whereas nodes 111 and 119 are located at the third layer. After conducting the experiment on the communication range of our proposed solution, the nodes are fixed at the same positions in Figure 11, from where they are responding reliably. To conclude the distance coverage test, the measured point-to-point distance is 250 m, which increases to nearly 750 m by adding two intermediate nodes. 

A long-term test was performed on the fixed poles in an open area of the grape farm. Each node was powered by 10 W monocrystalline solar panel and had a moisture sensor attached, which measured soil status in the area. The test was conducted for 5 days from 16 January 2020 to 20 January 2020. The data request time was set from 10:00 a.m. to 1:00 p.m. On a daily basis, the gateway node (Node 0) gets weather data (e.g., air temperature and humidity) from OpenWeatherAPI and generates soil moisture requests to nodes 1, 11, 111, and 112. A retry mechanism was also used at the gateway node to keep a daily record of the moisture status in different areas. During data collection on 2nd and 3rd day, water was poured on the zones of node 111 and 112, respectively.

**Figure 12:**
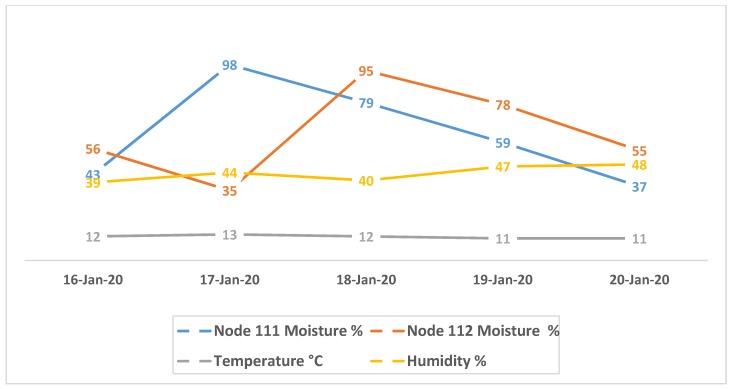
Historical soil moisture and weather data of node 111 and node 112.

As shown in Figure 12, the results indicate that the moisture level was sharply increased after pouring the water. Moreover, the air temperature and humidity have a strong correlation with soil moisture, therefore, these parameters have been used in the testing. For instance, the increase temperature will cause decrease in moisture percentage.

### 5.3. Network Performance Test

The maximum communication range of a single hop is 250 m in radius. The tests were performed with two and three hops, with each hop having a distance of 250 m. A router that was placed in between hops functioned as a repeater. It reconstructed the packet and forwarded it to the destination, thereby regenerating the radio signal. A series of tests was performed in the corridor within the line of sight, with a different packet size up to a maximum of 256 bytes at an underlying rate of 9.6 kbps. Our system supports next-hop routing for controlled messages in the tree and many-to one routing to the gateway. 

In this phase of the experiment, a real-time test was performed to check the latency and packet loss rate at different packet sizes. The test was performed during sunny weather from 10:00 a.m. to 5:00 p.m. The network consisted of four nodes located at three different layers. For each node, hundreds of packets were sent continuously at each packet size. Thus, a total of 400 packets were sent at each packet size from the gateway to the nodes. Packet loss and time delay were measured for each node in the network. The packet loss rate was slightly increased with packet size, but the per-node results in Table 2 show that the loss rate was not completely dependent on packet size, and other factors, such as power issue and antenna placement, could be involved. In the 16-byte test, the child node loss rate was less than that of its parent when a tree structure where the parent packet loss should reflect those of its child nodes was deployed. The reason for such finding is that sequential data requests are generated from the gateway node, and the gateway waits for the reply of the generating packet and increases the node ID in case of a packet reply or time-out. Arguably, node 11 became active when a packet was generated for node 111. The total packet loss in the network at different packet sizes is presented in Table 3.

**Figure 13:**
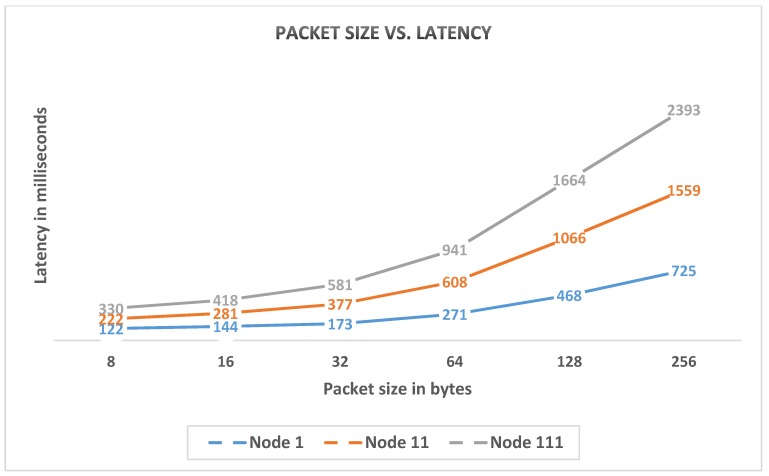
Network latency on different packet sizes.

Network time delay was also tested in a multi-hop scenario at different packet sizes. Given that our network mechanism is based on a tree structure, Figure 13 shows that latency increases by adding each intermediate node.

The acceptable latency and packet loss depend on the application. Table 4 presents the comparisons of latency, communication range, and packet loss between ZigBee [44] and our proposed module HC12 at a given packet size. Packet loss is nearly the same, but the communication range is considerably higher in our system, which is the most important aspect of our application. Although its network latency is remarkably higher than that of ZigBee, our application is still acceptable because the nodes only report a small amount of data once a day. The proposed module outperforms the existing approach.

### 5.4. Comparisons with Existing Technologies

With the rapid growth of IoT in different applications, devices or protocols with such feature are preferred because they can fit well to application requirements. In the agricultural context, coverage distance and cost effectiveness are more important than data rate. Soil property measurement, which is mostly performed on certain intervals, e.g., daily or weekly, eliminates the need for a high-speed network. In accordance with application requirements, the serial-based communication device called HC12 [41] is the most appropriate solution due to its lower deployment cost and better communication range. Nevertheless, it has a low data rate with high latency, which is less important in our application. Table 5 presents the detailed comparison of the proposed module with other existing approaches based on several parameters, such as power consumption, communication range, deployment cost, and data rate.

## 6. Conclusions

This work proposes an information monitoring approach for collecting field data over long distances, which can be used in a fully automated agricultural farm. In this system, a network mechanism for HC12 module is designed to enhance the communication range. An experiment in an actual farm shows that the system performs better on soil measurement in a wide area. The test cases indicate that the system runs stably and accurately. As indicated in the results, the point-to-point distance is 250 m, which increases to 750 m when two intermediate nodes are added. The proposed approach supersedes existing works in terms of lower deployment cost and better communication range. Network performance is somehow better than existing approaches, except for latency, which is not a requirement for the target application. A long-term test is performed to check the real-time collection of field data, which results in accurate field status updates. Lastly, the proposed approach can be used as a reference model for any type of wide-area information monitoring system.

The transmission time delay can be considered a limitation factor if the system is implemented in applications where data speed is equally important. Nevertheless, low cost and distance coverage are more important than data speed in the agricultural context. 

The proposed system uses a single wireless channel for the entire network communication. Multi-channelization can be applied by dividing the network into clusters. Each cluster will use two different channels: one for the gateway and one for its child nodes. By implementing multi-channelization, the gateway will no longer wait for each node response; hence, delay time at the gateway can be considerably decreased. The limitation of sensor node failure due to a static parent node can also be eliminated by using a mesh scenario wherein each node will have a multipath toward its parent node. Lastly, system scalability on a large network can also be checked using a simulator.

## Figures and Tables

**Table 1:** Custom network packet.

Field Type	Number of Bytes
Packet ID	2 bytes
MAC address	2 bytes
Source address	2 bytes
Destination address	2 bytes
Data	N bytes

**Table 2:** Packet loss per node at different packet sizes.

Packet Size	Node 1	Node 11	Node 111	Node 112
8 bytes	2	2	4	4
16 bytes	1	4	3	5
32 bytes	1	2	4	6
64 bytes	2	3	6	4
128 bytes	1	5	6	4
256 bytes	2	3	7	9

**Table 3:** Network packet loss at different packet sizes.

Packet Size (Bytes)	Number of Packet Loss
8	12
16	13
32	13
64	15
128	16
256	21

**Table 4:** Network performance comparisons between HC12 and ZigBee.

Module	Packet Size	Distance	RTT	Packet Loss
ZigBee	50 bytes	85 m	18.6 ms	1.65%
HC12	64 bytes	250 m	271 ms	2%

**Table 5:** Detailed comparisons of the proposed module with existing technologies.

Parameters	GSM	ZigBee	LoRa	HC12
**Data Rate**	9600–115,200 b/s	20,000–250,000 b/s	300–50,000 b/s	1200–115,200 b/s
**Range**	-	10–100 m	5 km (URBAN), 15 km (RURAL)	200–1000 m
**Topology**	Distributed	Star/Mesh	Star on Star	Star on Star
**Transmit Power (max)**	29–39 dBm	3–4 dBm	20 dBm	20 dBm
**Spectrum Cost**	Subscription required	Free	Free	Free
**End Device Cost**	10–13 US$	20–23 US$	5–7 US$	2.5–3 US$
**Gateway Cost**	-	20–25 US$	100–150 US$	20–25 US$
